# Camptothecin induces G_2_/M phase arrest through the ATM-Chk2-Cdc25C axis as a result of autophagy-induced cytoprotection: Implications of reactive oxygen species

**DOI:** 10.18632/oncotarget.24934

**Published:** 2018-04-24

**Authors:** Rajapaksha Gedara Prasad Tharanga Jayasooriya, Matharage Gayani Dilshara, Ilandarage Menu Neelaka Molagoda, Cheol Park, Sang Rul Park, Seungheon Lee, Yung Hyun Choi, Gi-Young Kim

**Affiliations:** ^1^ Department of Marine Life Sciences, Jeju National University, Jeju 63243, Republic of Korea; ^2^ Department of Molecular Biology, College of Natural Sciences and Human Ecology, Dongeui University, Busan 47340, Republic of Korea; ^3^ Department of Biochemistry, College of Oriental Medicine, Dong-Eui University, Busan 47227, Republic of Korea; ^4^ Present address: Department of Bioprocess Technology, Faculty of Technology, University of Rajarata, Mihintale 50300, Sri Lanka

**Keywords:** camptothecin, G_2_/M phase arrest, ROS, ATM, Chk2

## Abstract

In the present study, we report that camptothecin (CPT) caused irreversible cell cycle arrest at the G_2_/M phase, and was associated with decreased levels of cell division cycle 25C (Cdc25C) and increased levels of cyclin B1, p21, and phospho-H3. Interestingly, the reactive oxygen species (ROS) inhibitor, glutathione, decreased CPT-induced G_2_/M phase arrest and moderately induced S phase arrest, indicating that the ROS is required for the regulation of CPT-induced G_2_/M phase arrest. Furthermore, transient knockdown of nuclear factor-erythroid 2-related factor 2 (Nrf2), in the presence of CPT, increased the ROS’ level and further shifted the cell cycle from early S phase to the G_2_/M phase, indicating that Nrf2 delayed the S phase in response to CPT. We also found that CPT-induced G_2_/M phase arrest increased, along with the ataxia telangiectasia-mutated (ATM)-checkpoint kinase 2 (Chk2)-Cdc25C axis. Additionally, the proteasome inhibitor, MG132, restored the decrease in Cdc25C levels in response to CPT, and significantly downregulated CPT-induced G_2_/M phase arrest, suggesting that CPT enhances G_2_/M phase arrest through proteasome-mediated Cdc25C degradation. Our data also indicated that inhibition of extracellular signal-regulated kinase (ERK) and c-Jun N-terminal kinase (JNK) inhibited CPT-induced p21 and cyclin B1 levels; however, inhibition of ERK blocked CPT-induced G_2_/M phase arrest, and inhibition of JNK enhanced apoptosis in response to CPT. Finally, we found that CPT-induced G_2_/M phase arrest circumvented apoptosis by activating autophagy through ATM activation. These findings suggest that CPT-induced G_2_/M phase arrest through the ROS-ATM-Chk2-Cdc25C axis is accompanied by the activation of autophagy.

## INTRODUCTION

Cell cycle checkpoints are important machines in eukaryotic cells that accurately regulate cell division by monitoring defects in the cell cycle [1]. Thus, the checkpoints permit uncontrolled cells to repair DNA damage or to consequently die by blocking cell division after DNA damage [2]. In particular, ataxia telangiectasia-mutated (ATM) and ATM and Rad3 related (ATR) protein kinases induce cell cycle delay in response to genotoxic stress, including reactive oxygen species (ROS) and chemicals, by inducing phosphorylation of checkpoint kinase 1 (Chk1) and Chk2 [3]. For instance, Chk1 is phosphorylated at Ser^317^ and Ser^345^ by ATM, and subsequently, autophosphorylated Ser^296^ in response to various types of DNA damage that can be induced by ionizing radiation. Phosphorylated Chk1 then prevents damaged cells from entering mitosis by inactivating Cdc25 phosphatases or by directly abrogating mitotic spindle formation through the activation of aurora B and BubR1 [4]. Chk2 is also activated by ATM in response to single- or double-strand DNA breaks and, consequently, targets Cdc25C phosphatase by stabilizing p53, which upregulates the activation of cyclin-dependent kinase 2 (Cdk2) and cyclin B1 to facilitate G_2_/M phase cell cycle arrest [5, 6]. Therefore, ATM-mediated Chk1 and Chk2 act at the cell cycle checkpoints by generating ROS to determine whether the damaged cells are repaired, or whether they undergo G_2_/M phase arrest or apoptosis. This suggests that the ROS-ATM-Chk1/2 route is a promising therapeutic strategy as a drug-target for cancer [7].

Nrf2 is a transcription factor that activates an antioxidant response that inhibits ROS formation and induction of Nrf2-related genes is imperative for defective cells to counteract ROS-induced oxidative damage [8]. Additionally, Nrf2 disruption causes oxidant-induced acute lung injury and inflammation in mice, and Nrf2-knockout mice are greatly predisposed to chemical-induced DNA damage [9, 10]. A previous study reported that inhibition of Nrf2 by excessive ROS formation caused DNA damage, specifically single- or double-strand DNA breaks, leading to the activation of ATM [11]. Upon ROS production, the activation of cell cycle checkpoints in response to oxidative damage-induced ATM phosphorylation is also essential for maintenance of genomic integrity and tumor suppression [12]. In particular, several kinds of cyclin and cyclin-dependent kinase (Cdk) complexes are implicated in checkpoint surveillance induced by single- or double-stranded DNA breaks and that activates cell cycle progression. The cyclin D-Cdk4/6 complex is active in early G_1_ phase, whereas the cyclin E-Cdk2 complex is required for entry into S phase, and the cyclin B1-Cdk1 complex is required for mitosis [13]. After DNA damage is induced by oxidative stress, p21-induced cell cycle arrest is also activated during DNA repair, and p21 inhibits Cdk which regulates many cellular processes in a p53-depedent and independent manner [14]. A recent publication also showed that p21 protected cells against oxidative stress through the upregulation of Nrf2, by competitively interacting with motifs in Nrf2 to compete with Keap1-Nrf2 binding, and by compromising ubiquitination of Nrf2 [15]. Therefore, ROS-induced Nrf2 may be an important method for regulating the cell cycle and apoptosis.

Camptothecin (CPT) specifically inhibits eukaryotic DNA topoisomerase I (topo I) by trapping a covalent enzyme-DNA intermediate [16]. Zeng *et al.* showed that CPT enhanced apoptosis in cancer cells by targeting the 3′-untranslated regions (UTR) of Bak1, p53, and Mcl1 through microRNA-125b-induced mitochondrial pathways [17]. Park *et al.* reported that CPT promotes Cdc2 and cyclin E-associated kinase activities in response to DNA damage [18]. Huang *et al.* suggested that CPT-induced single-strand DNA breaks are differentially involved in homologous recombination repair by Chk1 and Chk2 [19]. Nevertheless, there have been no reports addressing whether CPT induces G_2_/M phase cell cycle arrest through ROS/Nrf2-induced ATM activation, and, in turn, autophagy-induced cytoprotection. In this study, we found that CPT induced an irreversible G_2_/M phase cell cycle arrest in LNCaP cells through ROS-induced ATM-Chk2-Cdc25C and activation of extracellular-signal regulated kinase (ERK) and c-Jun-N-terminal kinase (JNK). Furthermore, we found that CPT-induced autophagy protects cells from apoptosis and directs G_2_/M phase cell cycle arrest.

## RESULTS

### CPT irreversibly induces G_2_/M phase arrest in multiple cancer cell lines

**Figure 1:**
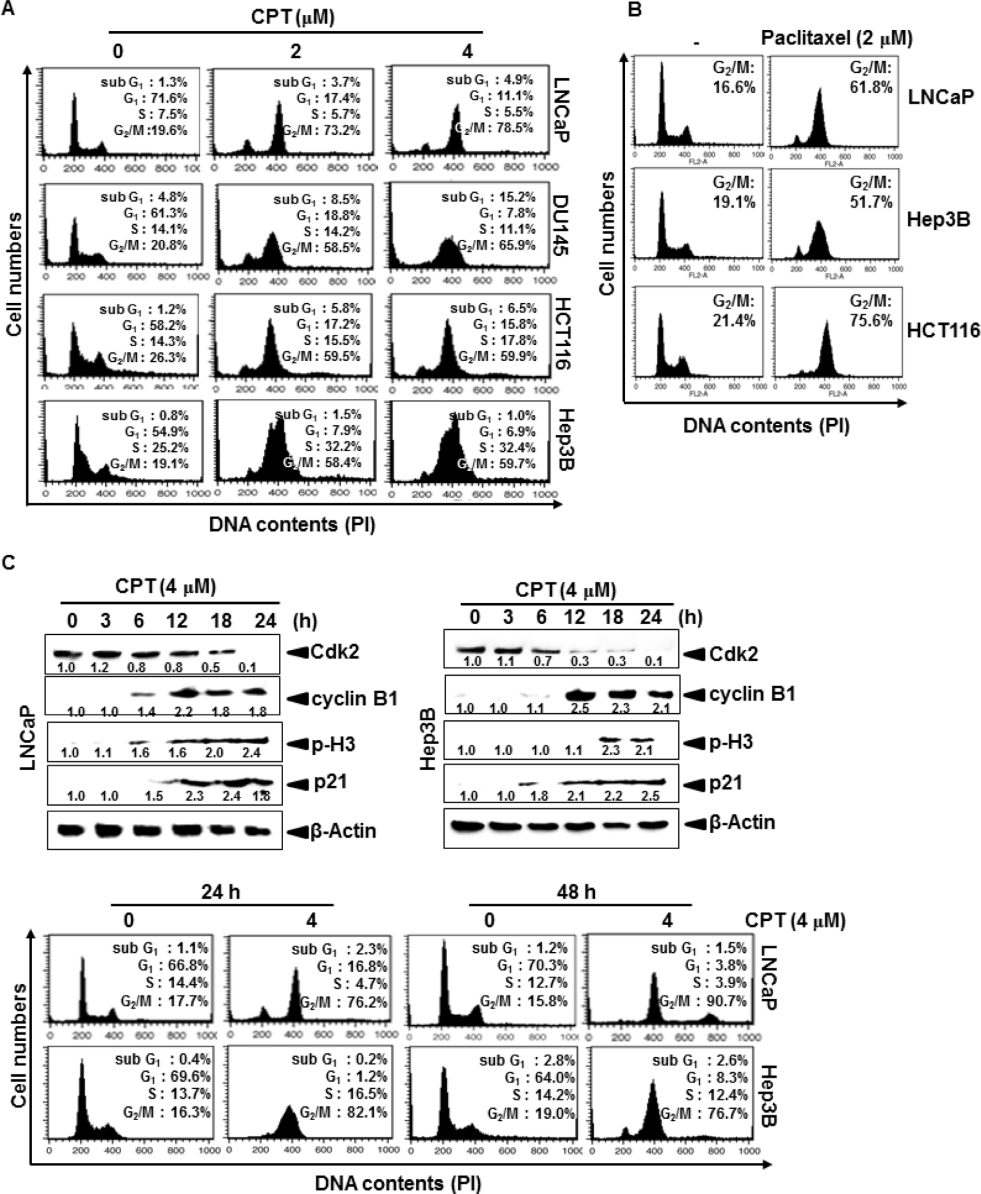
Camptothecin (CPT)-induced G_2_/M phase arrest Cells were seeded at 1 × 10^5^ cells/ml and were treated with CPT (2 μM and 4 μM) and paclitaxel (2 μM) for 24 h. (**A** and **B**) Cells were harvested, stained with propidium iodide, and analyzed to determine the cell cycle stage. (**C**) LNCaP cells and Hep3B cells were treated with 4 μM CPT for the indicated time points. Cell extracts were prepared for western blot analysis to examine cyclin B1, p-H3, p21, and Cdk2. (**D**) Cells were treated with 4 μM CPT for 24 h and 48 h, and the cells subsequently were processed for analysis for cell cycle distribution or the remaining cells were cultured in drug free culture media for another 24 h and cell cycle distribution was checked. Data from three independent experiments are presented.

CPT was previously showed to inhibit tumor cell growth by inducing apoptosis via a mitochondrial-dependent pathway [17]; however, the mechanism by which CPT contributes to cell cycle progression has not been described in detail. Therefore, we first examined the effect of CPT on cell cycle distribution using propidium iodide. Treatment with CPT significantly increased the number of G_2_/M phase cells at 24 h, which was accompanied by a decrease in the number of G_0_/G_1_ phase cells in LNCaP, DU145, HCT116, and Hep3B cells (Figure 1A). Treatment with 4 μM CPT strongly induced G_2_/M phase arrest, causing 55% of treated cells to arrest in all cell lines. Additionally, the sub-G_1_ population, which indicates apoptotic cell death, slightly increased in DU145 and HCT116 cells. CPT-induced G_2_/M phase arrest is similar pattern to the treatment of paclitaxel (Figure 1B). To further evaluate CPT-induced G_2_/M phase arrest, we examined changes in the expression of proteins that control cell cycle transition in LNCaP and Hep3B cells. As shown in Figure 1C, a gradual decrease in Cdk2 expression suggested that treatment with CPT moves the cells from G_1_/S phase to G_2_/M phase, because Cdk2 is most active in the S phase and decreases in G_2_/M phase. Our data also confirmed that CPT-induced G_2_/M phase arrest was accompanied by p21 and cyclin B1 expression, which functions as a tumor suppressor and initiates cell cycle arrest by inhibiting Cdk activity in G_2_/M phase in response to DNA damage [20]. Additionally, treatment with CPT resulted in a significant increase in p-H3 expression, which is a crucial event in the onset of mitosis [2]. Finally, to determine whether CPT-induced G_2_/M phase arrest was irreversible, the cells were treated with CPT for 24 h, moved to CPT-free media, and then examined for cell cycle distribution at the indicated times. Treatment with CPT increased the number of cells in G_2_/M phase arrest at 24 h, and the arrest was sustained when cells were incubated in CPT-free media for an additional 24 h (Figure 1D), indicating that CPT irreversibly induces G_2_/M phase arrest. Taken together, these results indicate that CPT irreversibly induces G_2_/M phase arrest in multiple cancer cell lines, which is accompanied by a change in the expression of G_2_/M phase-regulating checkpoint proteins.

### ROS are the potential mechanism of CPT-induced G_2_/M phase arrest

**Figure 2:**
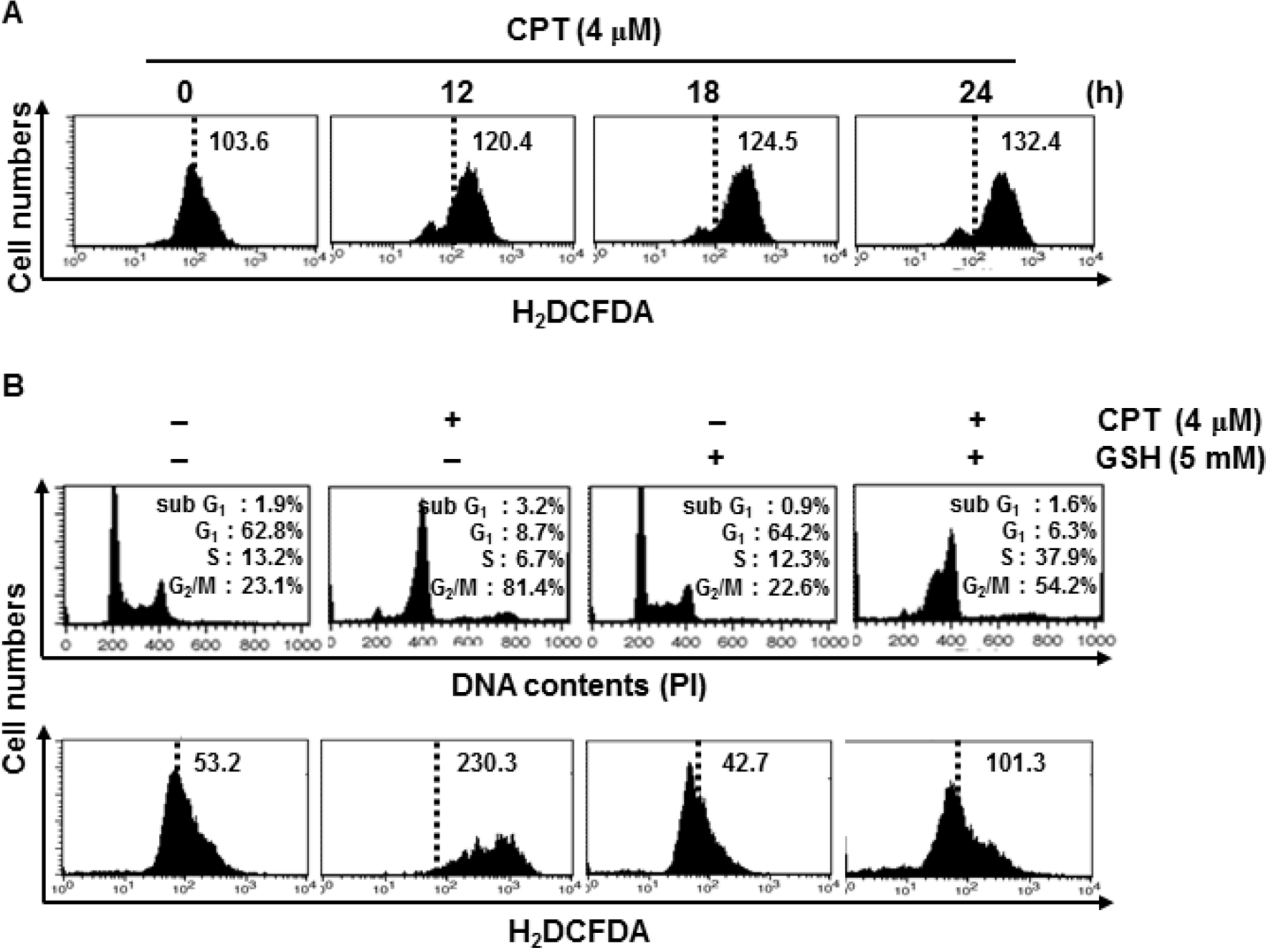
Camptothecin (CPT)-induced reactive oxygen species (ROS) mediate G_2_/M phase cell cycle arrest (**A**) Effect of CPT on ROS production. LNCaP cells were treated with 4 μM CPT for the indicated time points. H_2_DCFDA-based fluorescence detection was performed by flow cytometry. (**B**) LNCaP cells were treated with 5 mM glutathione for 30 min and then incubated with 4 μM CPT for 24 h. Cell cycle distribution was analyzed by flow cytometry after cells stained with propidium iodide. Cells were similarly treated, stained with H_2_DCFDA-based fluorescence, and monitored using flow cytometry.

A recent publication showed that chemical oxidant-induced intracellular ROS accumulation led to DNA damage and, consequently, induced cell cycle arrest by activating cell cycle checkpoints [21]. Therefore, we monitored ROS formation in CPT-treated LNCaP cells using H_2_DCFDA, which oxidizes in the presence of ROS. CPT significantly induced ROS formation in LNCaP cells in a time-dependent manner (Figure 2A). Next, we analyzed the level of ROS formation and the cell cycle distribution in the presence of the ROS inhibitor, glutathione (GSH). Treatment with CPT significantly increased G_2_/M phase arrest of approximately 80% of cell population (Figure 2B, top) with high ROS formation (Figure 2B, bottom), whereas pretreatment with GSH inhibited the CPT-induced G_2_/M phase arrest to approximately 55% of the cell population, while increasing the percentage of S phase cells with low levels of ROS. This result indicated that GSH could not completely inhibit CPT-induced G_2_/M phase arrest, but delayed the cell cycle or prevented S phase (Figure 2B). These data indicate that ROS are important factors in CPT-induced G_2_/M phase arrest.

### CPT-induced Nrf2 delays the cell cycle at S phase

**Figure 3:**
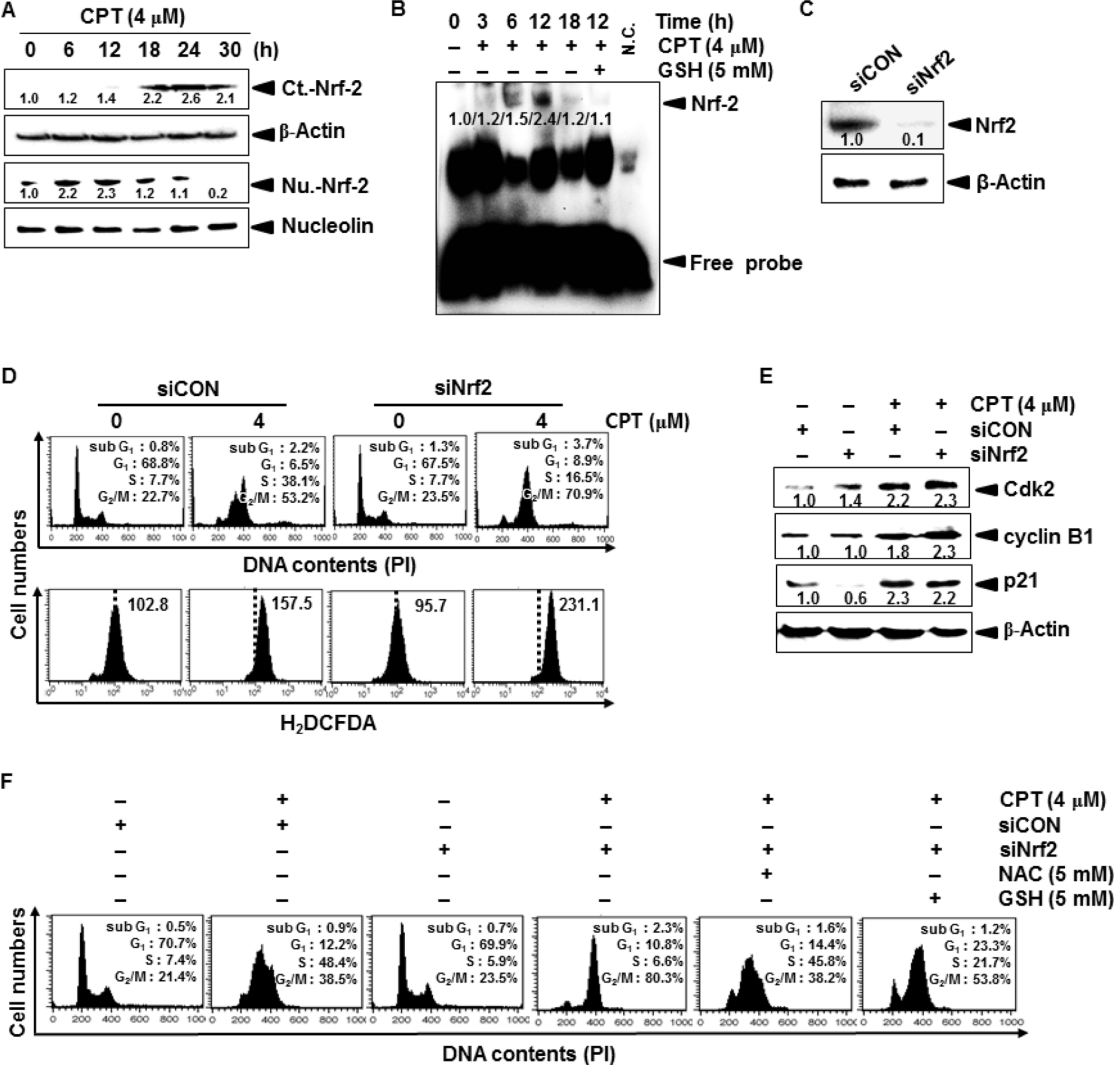
Camptothecin (CPT)-induced the expression of Nrf2 (**A**) LNCaP cells were incubated with 4 μM CPT for the indicated time points. Cytosol and nuclear lysates were resolved on SDS-polyacrylamide gels, transferred to nitrocellulose membranes, and probed with antibodies against Nrf2. (**B**) LNCaP cells were pretreated with NAC (5 mM) for 2 h and then incubated with CPT (4 μM) for indicated time points. Nuclear extracts were prepared to analyze ARE-binding of Nrf2 by EMSA. (**C**) Cells were transiently transfected with *Nrf2*-targeted siRNA (siNrf2) or with control siRNA (siCON), and then Nrf2 expression was examined by western blot analysis. (**D**) At 12 h, representative histograms for the effect of CPT treatment on cell cycle distribution in LNCaP cells transfected with siNrf2 or with siCON (*top*). A parallel experiment was used to measure ROS formation by staining cells with H_2_DCFDA-based fluorescence under similar condition (*bottom*). (**E**) Effect of siRNA-based Nrf2 protein depletion on cell cycle proteins. LNCaP cells were incubated with 4 μM CPT for the indicated time. Cytosol and nuclear lysates were resolved on SDS-polyacrylamide gels, transferred to nitrocellulose membranes, and probed with antibodies against p21, Cdk2, and cyclin B1. β-Actin was used as an internal control for western blot analysis. (**F**) Effect of siNrf2 on cell cycle distribution in the presence of the ROS inhibitors, NAC and GSH. LNCaP cells were transiently transfected with siNrf2 for 24 h and then treated with 5 mM NAC and 5 mM GSH for 1 h prior to incubation with 4 μM CPT for 12 h. Representative histograms for the effect of CPT treatment on cell cycle distribution as determined by flow cytometry.

Nrf2 activates an anti-oxidant response that counteracts ROS to protect against cellular damage [8]. Therefore, we tested whether Nrf2 delays CPT-induced G_2_/M phase arrest by inhibiting ROS formation. Western blot analysis indicated that CPT treatment gradually increased Nrf2 expression in the cytosolic compartment of LNCaP cells at 18 h; however, nuclear translocation of Nrf2 significantly increased at 6 h and was suppressed at 18 h (Figure 3A). EMSA also confirmed that CPT gradually induced the specific DNA-binding activity of Nrf2 at an early stage (at 6 h and at 12 h) after CPT treatment, and that the Nrf2 activity completely decreased at 18 h (Figure 3B). These results indicated that Nrf2 naturally alleviates ROS-induced oxidative damage at the early stages; however, Nrf2 was markedly downregulated at the late stages of CPT-induced ROS formation. Similar to CPT-induced downregulation of Nrf2, GSH significantly inhibited Nrf2-binding activity at the early stage. Next, we tested whether CPT-induced Nrf2 at the early stage (at 12 h) influences ROS-induced G_2_/M phase arrest. *Nrf2*-targeted siRNA (siNrf2) significantly reduced Nrf2 protein levels when compared to that of the control siRNA (siCON, Figure 3C). In a parallel experiment, siNrf2 significantly increased CPT-induced G_2_/M phase arrest (approximately 53% to 70%), and was accompanied by high levels of ROS formation, when compared to that of CPT treatment alone; however, siCON delayed the cell cycle at S phase, in CPT-treated cells at 12 h, suggesting that Nrf2 delays the cell cycle at S phase to allow cells to slowly move into G_2_/M phase (Figure 3D). As expected, western blot analysis showed that siNrf2 sustained CPT-induced cyclin B1 and p21 expression because siNrf2-treated cells proceeded to G_2_/M phase arrest. Surprisingly, Cdk2 sustained under the same condition, and the S phase cell population decreased in CPT-induced G_2_/M phase arrest in the presence of siNrf2 (Figure 3E). To confirm that CPT-induced G_2_/M phase arrest directly affected Nrf2 at the early stage (at 12 h) of ROS formation, we analyzed cell cycle distribution in the presence of the ROS inhibitors, *N*-acetyl-L-cysteine (NAC) and GSH. As shown in Figure 3F, CPT/siNrf2 significantly increased G_2_/M phase cell cycle arrest by approximately 80%; however, NAC and GSH markedly decreased G_2_/M phase arrest by approximately 40% and 50%, respectively, and increased the percentage of cells in S phase (Figure 3F). Taken together, these results indicate that CPT induces ROS-induced G_2_/M phase arrest, which was delayed in the presence of Nrf2.

### ATM-induced Chk2 is a key checkpoint in CPT-induced G_2_/M phase arrest through ROS formation

**Figure 4:**
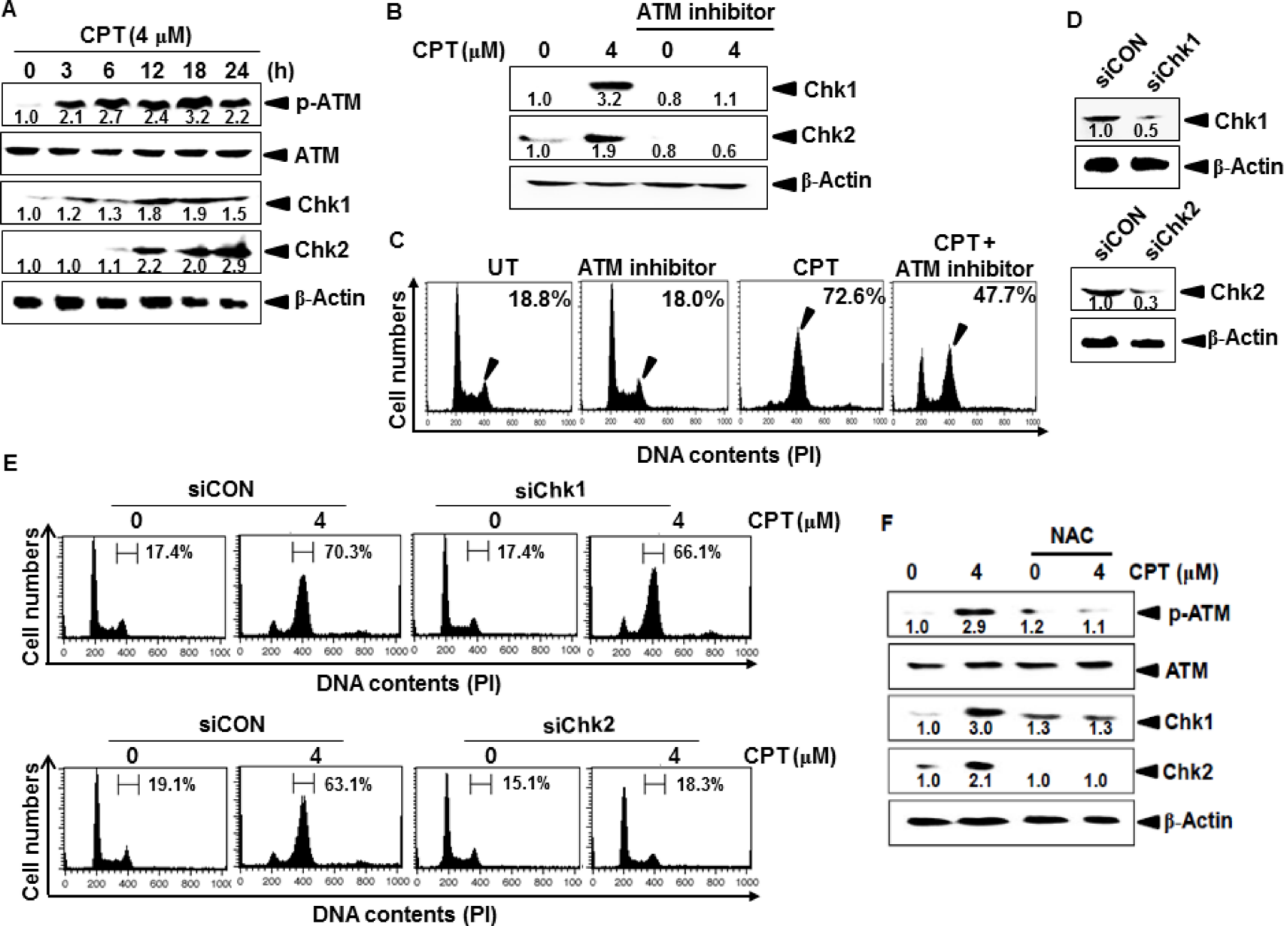
Effect of camptothecin (CPT) on ATM-induced Chks activation in LNCaP cells LNCaP cells were cultured in the presence of 4 μM CPT for 24 h. (**A**) Western blot analysis of the effects of CPT on levels of phosphorylated ATM and Chk1/2. (**B**–**C**) Effect of the ATM inhibitor on CPT-induced Chk1/2 expression. LNCaP cells were pretreated with the ATM inhibitor prior to incubation with 4 μM CPT for 24 h. Total protein was subjected to 10% SDS-PAGE followed by western blot analysis with antibodies specific for phosphorylated forms of Chk1 and Chk2 (B). In a parallel experiment, the cells were harvested, stained with propidium iodide and analyzed for cell cycle distribution (C). (**D**) Transient knockdown of Chk1 and Chk2. *Chk1*- and *Chk2*-targeted siRNA (siChk1 and siChk2) were transiently transfected into LNCaP cells for 48 h and then the expression levels of Chk1 and Chk2 were analyzed by western blot analysis. (**E**) After LNCaP cells were transfected with siChk1 (*top*) and siChk2 (*bottom*) for 24 h and then treated with 4 μM CPT for additional 24 h. DNA content was analyzed by flow cytometry. (**F**) LNCaP cells were pretreated with NAC (5 mM) for 1 h and then incubated with CPT (4 μM). ATM phosphorylation and expression of Chk1 and Chk2 were measured by western blot analysis. β-Actin was used as an internal control.

ATM is an upstream kinase implicated in the phosphorylation and activation of Chk1 and Chk2, and activated in response to genotoxic stress-induced cellular senescence through the DNA-damage response pathway [22]. Western blot analysis indicated that ATM phosphorylation increased in CPT-treated LNCaP cells in a time-dependent manner, and was accompanied by significant expression of downstream molecules of ATM, including Chk1 and Chk2 (Figure 4A). To further determine whether ATM activation directly activates Chk1- or Chk2-induced G_2_/M phase arrest, LNCaP cells were pretreated with an ATM inhibitor. Western blot analysis showed that CPT-induced Chk1 and Chk2 expression both were significantly inhibited in response to the ATM inhibitor (Figure 4B); the ATM inhibitor also abolished CPT-induced G_2_/M phase arrest (from approximately 72% to 47%) and increased G_0_/G_1_ phase cell populations (Figure 4C). These results suggested that ATM is a key regulator of CPT-induced G_2_/M phase arrest by activating Chk1 and Chk2. To verify the role of Chk1 and Chk2 in CPT-induced G_2_/M phase arrest, we used transient knockdown of *Chk1* and *Chk2* by siRNA (siChk1 and siChk2) to analyze cell cycle distribution. Western blot analysis indicated that transient transfection of siChk1 and siChk2 significantly reduced Chk1 and Chk2 protein levels when compared to those after transfection with siCON (Figure 4D). In further study, siChk1- and siChk2-tranfected cells were treated with CPT, and then the cell cycle distribution was assessed at 24 h. siChk1 in the presence of CPT did not affect the G_2_/M phase cell population when compared to that after of siCON transfectants (*top*); however, siChk2 significantly attenuated CPT-induced G_2_/M phase arrest (*bottom*), suggesting that Chk2 functions as a cell cycle checkpoint in CPT-induced G_2_/M phase arrest through ATM activation (Figure 4E). We also determined whether CPT-induced ROS formation activates ATM-induced Chk1 and Chk2 expression. As shown in Figure 4F, CPT significantly phosphorylated ATM; however, the presence of NAC markedly reduced the effect of CPT. These data indicate that CPT-induced G_2_/M phase arrest is influenced by the ATM-Chk2 axis.

### Cdc25C is required for proteasome-induced degradation in CPT-induced G_2_/M phase arrest

**Figure 5:**
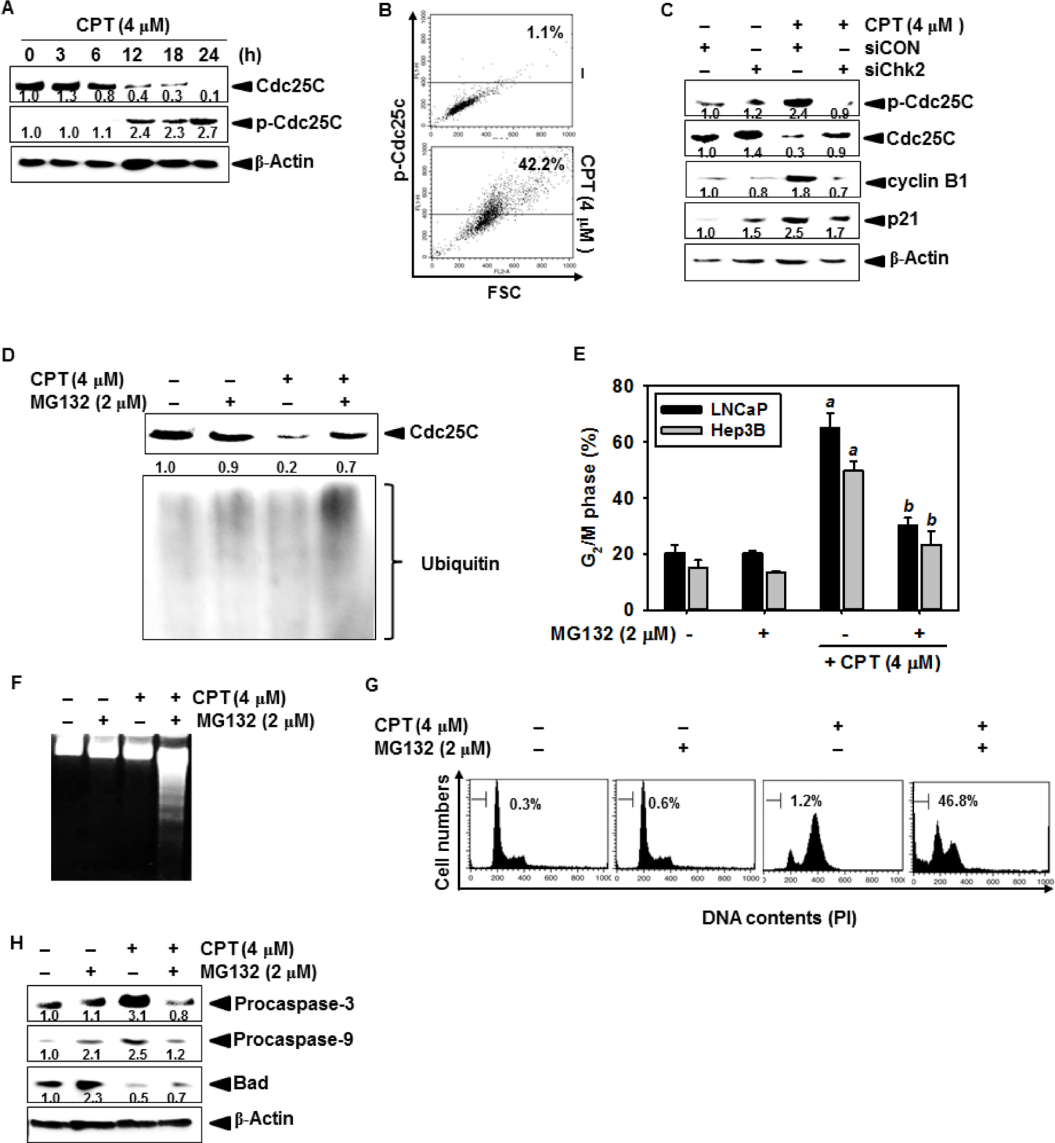
Camptothecin (CPT)-induced Cdc25C is degraded by the ubiquitin-proteasome pathway (**A**) Western blot analysis of Cdc25C levels using lysates from untreated control group and CPT-treated LNCaP cells. LNCaP cells were cultured in the presence of 4 μM CPT for 24 h. Total protein was subjected to 10% SDS-PAGE followed by western blotting with antibodies specific for phosphorylated forms of Cdc25C. (**B**) LNCaP cells were cultured in the presence of 4 μM CPT for 24 h. Cells were measured by dual analysis of p-Cdc25C and DNA content in control and CPT-treated cells. (**C**) Effect of Chk2 depletion on CPT-induced phosphorylation of Cdc25C and cell cycle protein. siChk2-transfected cells were treated with 4 μM CPT for 24 h, harvested and processed for western blot analysis using antibodies against Cdc25C, p-Cdc25C, cyclin B1, and p21. β-Actin was used as an internal control. (**D**) Effect of proteasome inhibitor MG132 on CPT-induced decline in Cdc25C protein levels. LNCaP cells were treated with 4 μM CPT in the presence or absence of MG132 for 24 h. Cell lysates prepared for western blot analysis using antibodies against ubiquitin to identify the high molecular weight polyubiquitin conjugates. (**E**) Effect of MG132 on CPT-induced cell cycle arrest. LNCaP and Hep3B cells were treated with 4 μM CPT in the presence or absence of MG132 prior to processing for analysis of cell cycle distribution. (**F**) Effect of treatment with combination of CPT and MG132 on DNA fragmentation. After treatment of LNCaP cells as indicated for 24 h, fragmented DNAs were extracted from the cells and analyzed on 1.5% agarose gels. (**G**) Cells with sub-G_1_ phase DNA content were detected by flow cytometry. The percentages of cells with sub-G_1_ DNA content are shown in each panel. (**H**) Effect of treatment with a combination of CPT and MG132 on levels of pro-apoptotic and anti-apoptotic protein. LNCaP cells were treated with 2 μM MG132 alone, 4 μM CPT alone, or a combination of both for 24 h. Cell extracts were prepared for western blot analysis for caspase-9, caspase-3, and Bad. β-Actin was used as an internal control. Data from three independent experiments are expressed as the overall mean ± S.E. Statistical significance was determined by one-way ANOVA (^*a*^ and ^*b*^, *p* < 0.05 vs. control and CPT-treated group).

Chk1 and Chk2 phosphorylate Cdc25C on Ser^216^ and consequently induce ubiquitination-induced degradation of Cdc25C, which is required to activate the mitotic kinases Cdc2/cyclin B1 to allow entry into G_2_/M [22]. As shown in Figure 5A, the level of Ser^216^-phosphorylated Cdc25C significantly increased after treatment with CPT; however, the total Cdc25C levels gradually decreased in response to CPT, which indicates that CPT induces phosphorylation-dependent Cdc25C degradation. Thus, we further examined the increases in p-Cdc25C expression using anti-p-Cdc25C antibody conjugated-FITC staining. Our results showed that CPT significantly increased the intracellular phosphorylation of Cdc25C at 24 h and increased cell size (toward high forward scatter) (Figure 5B), suggesting that CPT suppresses cytokinesis. Next, we determined whether Cdc25C is downregulated by CPT-induced Chk2 in LNCaP cells. The amount of CPT-induced Ser^216^-phosphorylation of Cdc25C was higher in siCON-transfected cells than in cells transfected with siChk2, and Cdc25C expression levels in siChk2 were normal compared to that of siCON-transfected cells (Figure 5C). Additionally, siChk2 downregulated CPT-induced cyclin B1 and p21, which indicate that siChk2 reduces the G_2_/M phase checkpoint proteins such as cyclin B1 and p21 by restoring Cdc25C expression (Figure 5C). To determine whether the expression and phosphorylation of Cdc25C are upregulated by increasing ubiquitination, we tested the functional effect of MG132, a specific proteasome inhibitor, on CPT-induced G_2_/M phase arrest. The decrease in CPT-induced Cdc25C protein levels was blocked in the presence of MG132 (Figure 5D); pretreatment with MG132 reversed CPT-induced G_2_/M phase arrest in LNCaP cells (approximately 20%) and Hep3B cells (approximately 10%) (Figure 5E), suggesting that CPT upregulates ubiquitination of Cdc25C in G_2_/M phase arrest. In a parallel experiment, we did not detect that apoptotic sub-G_1_ phase cells in response to CPT only; however, combined treatment with CPT and MG132 significantly increased DNA fragmentation, an apoptotic marker, in LNCaP cells (Figure 5F). Thus, we determined whether combined treatment with CPT and MG132 could synergistically induce apoptosis in LNCaP cells. As shown in Figure 5G, treatment of LNCaP cells with CPT and MG132 for 24 h significantly increased the accumulation of sub-G_1_ phase cells and substantially decreased the G_2_/M phase cell population. Additionally, treatment with CPT and MG132 decreased the levels of procaspase-3 and procaspase-9, leading to caspase-dependent apoptosis (Figure 5H). These data confirm that Chk2-induced Cdc25C degradation is required for CPT-induced G_2_/M phase arrest by activating proteasome pathway.

### JNK and ERK increase CPT-induced G_2_/M phase arrest and expression of cyclin B1 and p21

**Figure 6:**
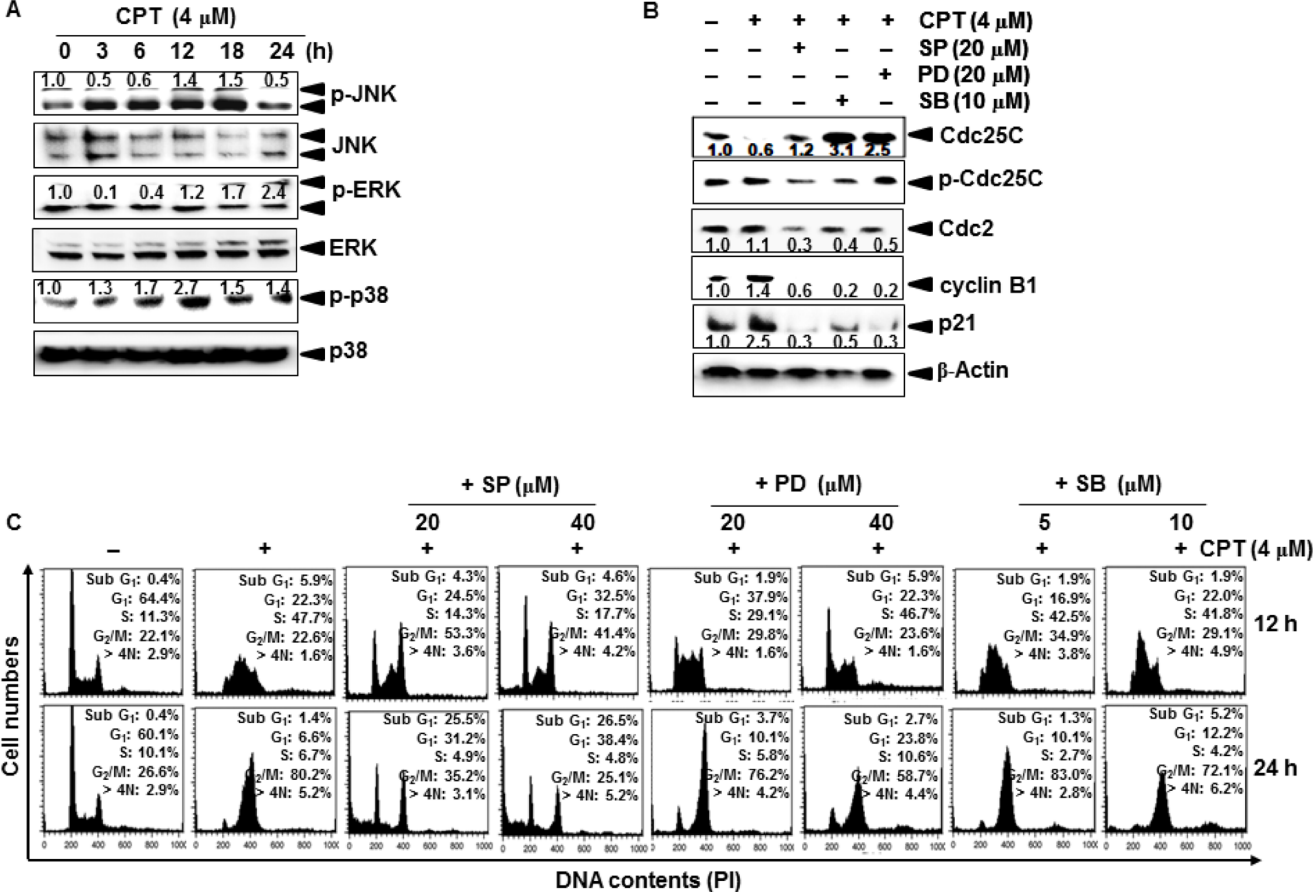
Camptothecin (CPT)-induced G_2_/M phase arrest through JNK and ERK activity (**A**) LNCaP cells were treated with 4 μM CPT for the indicated time points. Cell lysates were resolved on SDS-polyacrylamide gels, transferred to nitrocellulose membranes, and probed with antibodies against p-ERK, ERK, p-p38, p38, p-JNK, and JNK. (**B**) The LNCaP cells were stimulated with CPT 4 μM for indicated time points after pretreatment with 20 μM SP600125 (SP), 20 μM PD98059 (PD), and 10 μM SB203580 (SB) for 1 h. Cell lysates were resolved on SDS-polyacrylamide gels, transferred to nitrocellulose membranes, and probes with antibodies against Cdc25C, p-Cdc25C, cyclin B1, and p21. β-Actin was used as an internal control. (**C**) LNCaP cells were stimulated with 4 μM CPT for the indicated time after pretreatment with SP600125 (20 μM and 40 μM), PD98059 (10 μM and 20 μM), and SB203580 (5 μM and 10 μM) for 1 h. The cells were stained with PI and analyzed by flow cytometry at 12 h (*top*) and 24 h (*bottom*).

To determine whether mitogen-activated protein kinases (MAPKs) regulate cell cycle progression, we exposed LNCaP cells to CPT for different time points and measured MAPKs phosphorylation and activation. Our results showed that CPT increased the phosphorylation of JNK, ERK, and p38 at different time points (Figure 6A). Thus, we examined the expression of cell cycle proteins upon treatment with MAPK inhibitors. Pretreatment with a JNK inhibitor, SP600125, an ERK inhibitor, PD98059, and a p38 inhibitor, SB203580, decreased in decreasing CPT-induced expression of p21 and cyclin B1 (Figure 6B). Finally, we examined the functional effects of MAPKs on CPT-induced G_2_/M phase arrest at 12 h and 24 h. Treatment with CPT alone caused S phase arrest at 12 h and G_2_/M phase arrest at 24 h. However, pretreatment with SP600125 and PD98059 caused a greater decrease in S phase and G_2_/M phase cell populations at both time points; in particular, SP600125 significantly changed CPT-induced S phase arrest to G_2_/M phase arrest at 12 h and increased apoptotic sub-G_1_ phase population at 24 h, suggesting that JNK inhibition promotes CPT-induced G_2_/M phase arrest at the early stage, leading to apoptosis at the late stage (Figure 6C). Low concentration of PD98059 (10 μM) had no effect on the in response to CPT; however, 20 μM PD98059 completely blocked CPT-induced S phase arrest at 12 h, but had no effect at 24 h. However, SB203580 slightly affected CPT-induced S phase arrest and G_2_/M phase arrest. These data indicate that the ERK and JNK signaling pathways act through different molecular mechanisms to upregulate CPT-induced S phase arrest at 12 h and G_2_/M phase arrest at 24 h.

## DISCUSSION

**Figure 7:**
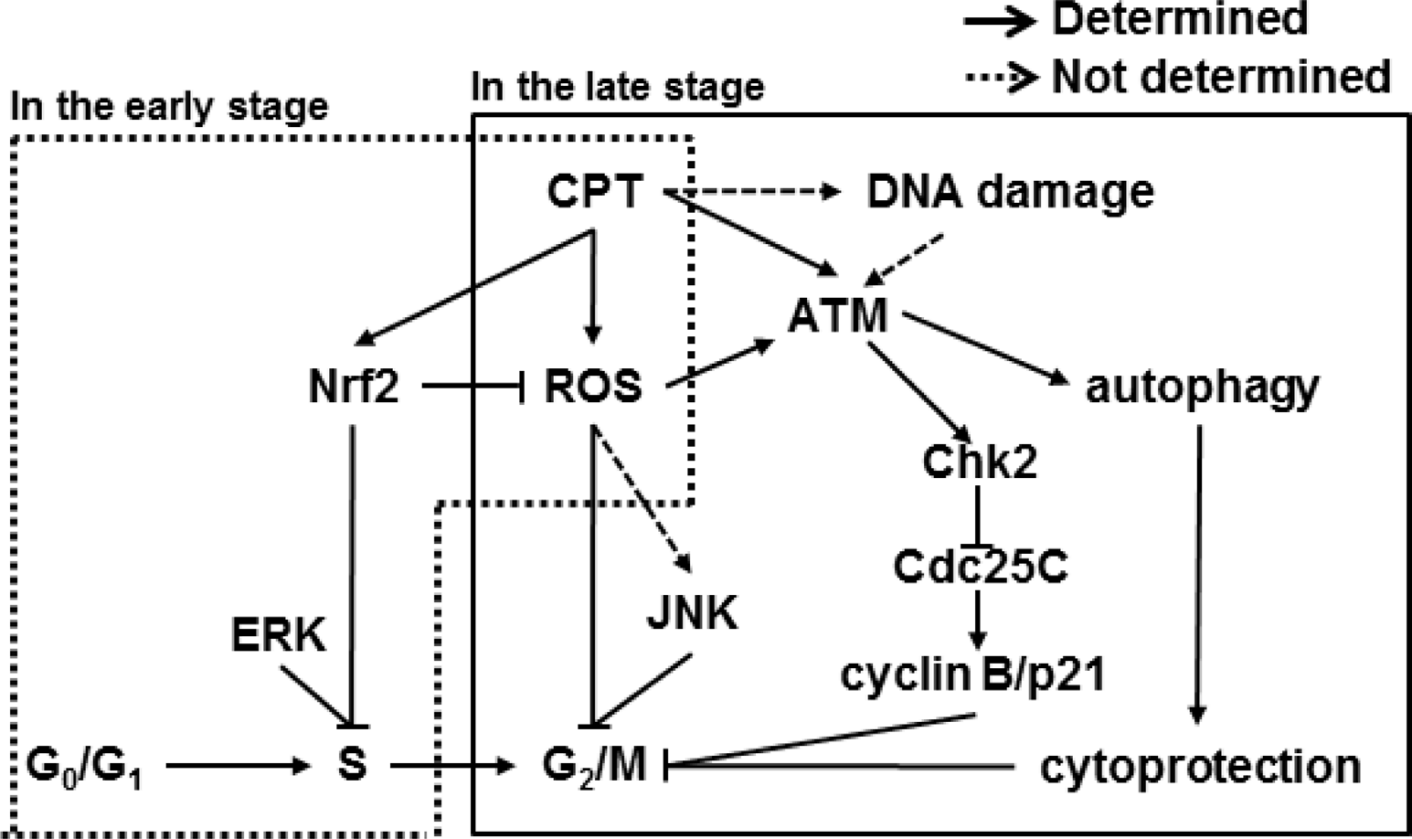
Scheme of camptothecin (CPT)-induced G_2_/M phase cell cycle arrest Model for the proposed CPT-induced cell cycle regulation. CPT-caused G_2_/M cell cycle arrest through activation of the ATM/Chk2/Cdc25C and ERK/JNK/cyclin B1 pathways. CPT-increased ROS formation and thus induced ATM phosphorylation, which, in turn, phosphorylated Chks. Activated Chk2 caused phosphorylation of Cdc25C. CPT also increased the phosphorylation of ERK and JNK to lead to increased levels of cyclin B1 and p21 expression, which induced S phase and G_2_/M phase arrest. Induction of ROS increased autophagy, which prevents apoptosis, directly inducing cell cycle arrest. ROS induction also enhanced Nrf2 translocation to activate the antioxidant genes and delays CPT-induced S phase.

Previous data confirmed that CPT forms a ternary complex with DNA and topo I which unwinds DNA during replication and transcription, and prevents cleaved DNA from rewinding [16]. Consequently, the ternary complex, topo I-CPT-DNA, causes S and G_2_ phase arrest-induced cytotoxicity by inducing DNA double-strand breaks [23]. Therefore, CPT was considered a promising candidate for treating malignant cancers; however, clinical use of CPT was limited because of poor solubility and adverse effects like myelosuppression, diarrhea, and hemorrhagic cystitis [24]. Nevertheless, CPT derivatives and analogues have been studied and designed for clinical practice because the unique ability of CPT to target topo I is attractive for the treatment of a broad spectrum of cancers [25, 26]. Therefore, we suggest that detail studies should be continued to determine the molecular action of CPT, which will help in the design of new topo I-targeting drugs with lower cytotoxicity. In the sense, we previously reported that CPT effectively inhibited phorbol myristate acetate (PMA)-induced invasion of prostate cancers by inhibiting matrix metalloprotease-9 (MMP-9) and vascular endothelial growth factor (VEGF) expression, which are downregulated by the upregulation of Nrf2-induced heme oxygenase-1 (HO-1), indicating that CPT suppresses cancer cell invasion without direct cytotoxicity [27]. Moreover, combined treatment with CPT and TNF-related apoptosis-inducing ligand (TRAIL) increased apoptotic cell death in human hepatocarcinoma Hep3B cells by upregulating death receptor 5 (DR5) expression. The upregulation occurs through ROS formation and the activation of ERK and of p38 MAPK, which suggests that subtoxic doses of CPT can be used as a TRAIL sensitizer to kill cancer [28]. Our previous studies suggest that CPT can be used for the treatment of cancer cells at low concentrations without causing cytotoxicity. Additionally, in this current study, we found that CPT induced S- and G_2_/M phase arrest by regulating cell cycle checkpoint proteins, resulting in autophagy (Figure 7).

Cell cycle checkpoints are central mechanisms in eukaryotic cells that control DNA replication, mitosis, and cytokinesis; however, harmful stresses potentially halt the cell cycle by activating checkpoint proteins. If this does not occur, the cells consequently die [3]. Therefore, cell cycle-targeting topo I inhibitors are fascinating pharmaceutics for treating cancers that act by, turning on the checkpoints. In particular, the topo I-targeting drugs are specific to the S phase [29]. In the current study, we first found that CPT sustained S phase arrest and induced Nrf2 at early stage (at 12 h). Transient knockdown of Nrf2 swiftly moved the S phase into G_2_/M phase arrest, suggesting that CPT-induced Nrf2 compromises S phase arrest at early stage. Although CPT potentially stopped S phase, because CPT targets topo I, which induces DNA double-strand breaks, CPT-induced S phase arrest transitioned into G_2_/M phase arrest at late stage (at 24 h). This result indicates that other factors activated by CPT-induced DNA breaks are involved in moving the cell into G_2_/M phase arrest. Surprisingly, inhibition of ROS formation allowed the transition of cells from CPT-induced G_2_/M phase arrest into S phase arrest, but not into the normal cell cycle distribution, which indicates that CPT induces the S phase arrest by inducing topo I inhibition-induced double-strand DNA breaks, leading to ROS formation, which influences G_2_/M phase arrest. However, questions remain about how ROS are generated and when ROS affect G_2_/M phase arrest. Kurz *et al.*, reported that ROS could induce topo I-induced oxidative DNA damage in an ATM-dependent manner, leading to cell death [30]. Recently, Ito *et al.*, found that ATM-deficient patients or ATM^-/-^ mice showed a significant increase in ROS formation, suggesting that impaired ATM function leads to defects in the control of ROS, regardless of DNA damage [6]. In contrast, in the current study, we showed that ROS and ATM are key checkpoint regulators in S and G_2_/M phase arrest, and that ROS inhibition suppressed ATM phosphorylation, which indicates that CPT-induced ROS are upstream initiators for activating ATM. Nevertheless, further studies will be necessary to determine whether double-stranded DNA break-induced ATMs or double-stranded DNA breaks itself themselves upregulate ROS formation and *vice versa*. Macip *et al.*, previously reported that p21 increased ROS levels in both normal and tumor cells [31], and Inoue *et al.* showed that transfection of p21 into LoVo and HCT116 cells triggered ROS levels in senescent or apoptotic cells [32]. In contrast, p21 directly binds to Nrf2 and blocks its ubiquitination-dependent degradation to stably reduce oxidative stress [15]. In the light of current study, p21-induced Nrf2 at early stage (at 12 h) attenuated oxidative damage through S phage arrest; however, accumulated ROS overcame antioxidant effect of Nrf2 at the late stage (at 24 h) and sustained p21 expression, which suggests that p21 may perform a dual function in S phase and G_2_/M phase. In the sense, we cannot also rule out the possibility that ROS act a different role at different time points by regulating cell cycle checkpoint proteins.

Recently, Schaar *et al.* [33] reported that mitochondrial and cytoplasmic ROS have reverse effects on lifespan, which indicates that cell cycle distribution and apoptosis could be also differently regulated, according to the compartments which promote ROS generation. In previous, CPT-mediated cell cycle arrest and death could be initiated by cytoplasmic ROS generated by NADPH oxidase [34] and scavenging mitochondrial ROS suppressed CPT-induced apoptosis [35, 36], suggesting that CPT simultaneously increases ROS generation from cytoplasm by NADPH oxidase and mitochondria by depolarization of its inner membrane, causing to cell death. Loza and Wellinger also found that CPT is capable of interacting with nuclear topo I, but mitochondrial topo I is not sensitive [37]. Then, we make one possibility that nuclear DNA damage targeted by CPT stimulates ROS generation from cytoplasm by NADPH oxidase, which precedes mitochondrial hyperpolarization, causing to ROS generation from mitochondria. Nevertheless, further researches will be required to evaluate robust function of CPT-mediated cytoplasmic and mitochondrial ROS on cell cycle distribution and death.

S phase and G_2_/M phase are tightly regulated by two checkpoint proteins, Chk1 and Chk2, which are activated by DNA damage-induced phosphorylation of ATM and ATR [32]. Chk1 is phosphorylated at Ser^345^ or Ser^317^ by ATM and ATR, and subsequently phosphorylates Cdc25A/C, leading to S or G_2_/M phase arrest [38]. Chk2 is activated by phosphorylation at Thr^68^ in an ATM-dependent manner [39]. Previous data also showed that Chk1 and Chk2 are differentially upregulated during cell cycle arrest in response to double strand DNA breaks induced by CPT, making that Chk1 inhibition is an attractive therapeutic strategy in CPT-driven DNA damage response [18]. Our data showed that CPT increased the expression of Chk1 and Chk2 in an ATM-dependent manner; however, transient knockdown of Chk2, but not Chk1, completely restored the cell cycle distribution from G_2_/M phase arrest. Additionally, S phase arrest was not observed in response to CPT after the knockdown of Chk1, suggesting that CPT-induced Nrf2, which inhibited the cell cycle distribution at S phase, is an upstream regulator of Chk2 because Chk2 broadly resides in S and G_2_/M phase. During genomic stress, the activation of Chks renders Cdc25C inactive via phosphorylation of Ser^216^ and ubiquitination-dependent degradation, thereby blocking the downstream signaling pathway involving p21 and cyclin B1 activation which is required for entry into G_2_/M phase [40]. Our data also indicated that Chk2 induced phosphorylation at Ser^216^ and ubiquitination-induced degradation of Cdc25C, which led to downregulation of p21 and cyclin B1, leads to CPT-induced G_2_/M phase arrest.

MAPKs are highly conserved serine/threonine protein kinases involved in a number of fundamental cellular processes, including environmental stress response, proliferation, differentiation, survival, and apoptosis. In particular, activation of the JNK and p38 pathways controls the apoptotic response induced by some DNA-damaging agents, whereas, activation of the ERK pathway is associated with proliferation and differentiation [41]. Under UV irradiation, JNK activation induced the phosphorylation of Cdc25C at Ser^168^ during DNA damage-induced G_2_/M phase arrest [42]. Furthermore, ERK is required to upregulate G_2_/M progression by disrupting cyclin B1-Cdc2 complex [43] and p38 MAPK increases the mitotic stage by activating Cdc25C [44]. These results indicated that MAPKs are essential to regulate cell cycle distribution; however, each MAPK responds differently to different stresses and drugs. In the current study, we found that JNK inhibition increased CPT-induced G_2_/M phase arrest and significantly decreased Cdc25C phosphorylation at Ser^216^, leading to apoptosis and ERK inhibition to surprisingly delay the route into G_2_/M phase arrest. p38 inhibition caused similar changes in the phosphorylation and expression of Cdc25c as those caused by JNK and ERK inhibition, but did not influence cell cycle distribution. Nevertheless, downstream molecules of Cdc25C, such as cyclin B1 and p21, decreased in response to all MAPK inhibitors, suggesting that JNK and ERK are involved in CPT-induced cell cycle distribution. Additionally, a recent study indicated that autophagy is a key player in the regulation of apoptosis and cell cycle, and, in DNA damage, checkpoint proteins enhance autophagy during mitosis by inducing MAPK activation, suggesting that autophagy delays the cell cycle to determine cell fate [45, 46]. Given the specific role of autophagy, MAPKs may regulate cell cycle distribution via autophagy. In this study, CPT inhibited cell proliferation, but not cell death; however, inhibition of autophagy moved CPT-induced G_2_/M phase arrest to apoptosis, which suggests that CPT-induced autophagy triggers cytoprotective effects, leading to G_2_/M phase arrest (Supplementary Figures 1 and 2). Nevertheless, we did not show direct interaction between MAPKs and autophagy; therefore, we need to further study how MAPKs, especially JNK and ERK, interplay between G_2_/M phase arrest and autophagy in response to CPT.

In conclusion, CPT promotes G_2_/M phase arrest and upregulates expression of Chk2 and Cdc25C, as a result of DNA damage-ROS-induced ATM phosphorylation; however, Nrf2 delays the cell cycle at the early stage. The JNK and ERK signaling pathways and autophagy are involved in CPT-induced G_2_/M phase arrest. These converging views may offer great opportunities for pharmacological intervention by CPT in cell cycle regulation and apoptosis.

## MATERIALS AND METHODS

### Reagents and antibodies

CPT, 3-(4,5-dimethylthiazol-2-yl)-2,5-diphenyltetrazolium bromide (MTT), propidium iodide, glutathione (GSH), *N*-acetyl-L-cysteine (NAC), MG132, 3-methyladenine (3MA), and bafilomycin A1 (BAF) were purchased from Sigma (St. Louis, MO) and an enhanced chemiluminescence (ECL) kit was purchased from Amersham (Arlington Heights, IL). RPMI 1640 medium, fetal bovine serum (FBS), and antibiotics mixture was purchased from WelGENE (Daegu, Republic of Korea). PD98059, SP600125, and SB239063 were purchased from Calbiochem (San Diego, CA). Antibodies against Cdk2, cyclin B1, p21, β-actin, Nrf2, nucleolin, phospho (p)-ATM (Ser^1981^), ATM, Chk1, Chk2, p-Chk2 (Thr^68^), Cdc25c, p-Cdc25C (Ser^216^), ubiquitin, procaspase-3, procaspase-9, Cdk2, Bad, Beclin-1, LC3, and Atg-7 were purchased from Santa Cruz Biotechnology (Santa Cruz, CA). Antibodies against p-histone (H)-3, p-ERK, ERK, p-p38, p38, p-JNK, and JNK were purchased from Cell Signal (Beverly, MA). Peroxidase-labeled donkey anti-rabbit and sheep anti-mouse immunoglobulin were purchased from Koma Biotechnology (Seoul, South Korea).

### Cell culture and viability assay

Human prostate cancer cell lines LNCaP and DU145, human hepatoma carcinoma cell line Hep3B, and human leukemia cancer cell line U937 were obtained from the American Type Culture Collection. Cells were cultured at 37° C in a 5% CO_2_-humidified incubator and maintained in RPMI 1640 medium containing 10% heat-inactivated FBS and 1% antibiotics mixture. The cells were seeded (4 × 10^4^ cells/ml) and then incubated with CPT for 24 h. MTT assays were performed to determine relative cell viability.

### DNA fragmentation assay

After treatment with CPT for 24 h, LNCaP cells were lysed in DNA fragmentation lysis buffer containing 10 mM Tris (pH 7.4), 150 mM NaCl, 5 mM EDTA, and 0.5% Triton ×-100 for 1 h on ice. Lysates samples were vortexes and separated centrifugation at 13,000 *g* for 15 min. Fragmented DNA in the supernatant was extracted with equal volume of phenol:chloroform:isoamyl alcohol (25:24:1) mixture and analyzed electrophoretically on 1.5 % agarose gels.

### Flow cytometric analysis

Cells were fixed in 1 U/ml of RNase A (DNase free) and 10 µg/ml propidium iodide overnight in the dark at room temperature. To assess whether apoptosis had occurred, the cells were incubated with annexin-V (R&D Systems). A FACSCalibur flow cytometer (Becton Dickinson, San Jose, CA) was used to determine the number of apoptotic cells, i.e., cells with sub-G_1_ DNA that were annexin-V^+^.

### Measurement of ROS

Cells were plated at a density of 5 × 10^4^ cells/ml, allowed to attach for 24 h, and exposed to 5 mM of NAC alone, 5 mM of GSH alone, 4 µM of CPT alone, or NAC or GSH plus CPT for 1 h. The cells were stained with 10 µM of H_2_DCFDA for 10 min at 37° C and flow cytometry was used to determine the fluorescence intensity.

### Western blot analysis

Whole-cell lysates were prepared by PRO-PREP protein extraction solution (iNtRON Biotechnology, Sungnam, Republic of Korea). Cytoplasmic and nuclear protein extracts were prepared using NE-PER nuclear and cytosolic extraction reagents (Pierce, Rockford, IL). The cell lysates were harvested from the supernatant after centrifugation at 13,000 *g* for 20 min. Total cell proteins were separated on polyacrylamide gels and standard procedures were used the transfer them the nitrocellulose membranes. The membranes were developed using an ECL reagent.

### Electrophoretic mobility shift assay (EMSA)

Transcription factor-DNA binding activity assays were carried out with nuclear protein extract. Synthetic complementary anti-oxidant response element (5′- TMANNRTGAYNNGCRWWWW-3′) binding oligonucleotides was 3′-biotinylated utilizing the biotin 3′-end DNA labeling kit (Pierce) according to the manufacturer’s instructions, and annealed for 30 min at 37° C. Samples were loaded onto native 4% polyacrylamide gels pre-electrophoresed for 60 min in 0.5X Tris borate/EDTA (TBE) buffer on ice, in the presence of transferred onto a positively charged nylon membrane (Hybond^™^-N+) in 0.5X TBE buffer at 100 V for 1 h on ice. The transferred DNA-protein complex was cross-linked to the membrane at 120 mJ/cm^2^. Horseradish peroxidase-conjugated streptavidin was utilized according to the manufacturer′s instructions to monitor the transferred DNA-protein complex.

### Small interfering RNA (siRNA)

Cells were seeded on a 24-well plate at a density of 1 × 10^5^ cells/ml and transfected *Nrf2*-, *Chk1*-, and *Chk2*-specific silencing RNA (siRNA, Santa Cruz Biotechnology) for 24 h. For each transfection, 450 μl of growth medium was added to 20 nM siRNA duplex with the transfection reagent G-Fectin (Genolution Pharmaceuticals Inc., Seoul, Republic of Korea) and the entire mixture was added gently to the cells.

### Statistical analysis

The images were visualized with Chemi-Smart 2000 (VilberLourmat, Marine,Cedex, France). Images were captured using Chemi-Capt (VilberLourmat) and transported into Photoshop. All bands were shown a representative obtained in three independent experiments and quantified by Scion Imaging software (http://www.scioncorp.com). Statistical analyses were conducted using SigmaPlot software (version 12.0). Values were presented as mean ± standard error (S.E.). Significant differences between the groups were determined using the unpaired one-way and two-way ANOVA test by Bonferroni′s test. Statistical significance was regarded at ^*a*^ and ^*b*^, *p* < 0.05.

## SUPPLEMENTARY MATERIALS FIGURES


