# Supplementary material for: Camptothecin induces G2/M phase arrest through the ATM-Chk2-Cdc25C axis as a result of autophagy-induced cytoprotection: Implications of reactive oxygen species
Source: Oncotarget. 2018 Apr 24;9(31):21744–57. doi: 10.18632/oncotarget.24934 (PMC5955160; doi:10.18632/oncotarget.24934)
Supplement: Supplementary file 1 [file oncotarget-09-21744-s001.pdf]

Camptothecin induces G_2/M phase arrest through the ATM-Chk2-Cdc25C axis as a result of autophagy-induced cytoprotection: Implications of reactive oxygen species

SUPPLEMENTARY MATERIALS

CPT-induced autophagy blocks cell death, leading to the promotion of G_2/M phase arrest

To determine the effect of CPT on cell viability and proliferation, human prostate cancer LNCaP and DU145 cells were treated with different concentrations of CPT and incubated for 24 h. Cell viability and growth were determined by MTT and trypan blue exclusion assays. As shown in Supplementary Figure 1A and 1B, CPT treatment significantly decreased cell viability and proliferation, respectively. However, apoptotic annexin-V^+ populations were not detected when LNCaP cells were treated with CPT for 24 h (Supplementary Figure 1C). Similarly, light microscopy showed that apoptotic shrinkage occurs in H_2O_2-treated LNCaP cells (Supplementary Figure 1D). These data indicate that CPT inhibits proliferation of LNCaP cells, but does not induce apoptosis.

Since CPT did not induce apoptotic death in LNCaP cells, we examined the effect of CPT on the induction of autophagy. Western blot analysis showed that CPT significantly induced the expression of the autophagy-related proteins, beclin-1, active LC3-II, and Atg-7, in a dose-dependent manner (Supplementary Figure 2A) at 24 h and in a time-dependent manner (Supplementary Figure 2B), indicating that CPT promotes the expression of autophagy proteins in LNCaP cells. Furthermore, we examined whether autophagy regulates CPT-induced inhibition of cell viability. For the functional study of autophagy, we pretreated cells with 3-methyladenine (3MA) which inhibits LC3-I to LC3-II, and bafilomycin A1 (BAF), which blocks the fusion of autophagosomes and lysosomes in the presence of CPT. As sown, 3MA (0.6 mM) and BAF (20 nM) slightly decreased CPT-induced inhibition of cell viability (Supplementary Figure 2C). Next, we determined whether autophagy-mediated decrease of cell viability was due to apoptosis in the presence of CPT. Our data indicated that combined treatment with CPT and 3MA significantly increased the annexin-V^+ cell populations, indicating that CPT-induced autophagy blocks the move into apoptotic death (Supplementary Figure 2D). We also found that the ATM inhibitor suppressed CPT-induced Beclin-1 and LC3-II expression, suggesting that CPT-induced ATM promotes autophagy, resulting in protection against apoptosis (Supplementary Figure 2E). Western blot analysis also confirmed that CPT decreased procaspase-3 and increased proapoptotic Bad in response to 3MA in human leukemia U937 cells (Supplementary Figure 2E). Finally, 3MA-mediated inhibition of autophagy gradually increased DNA fragmentation in response to CPT in human leukemia U937 cells (Supplementary Figure 2F). Taken together, these results indicate that CPT-induced autophagy blocks cell death and increases G_2/M phase arrest.

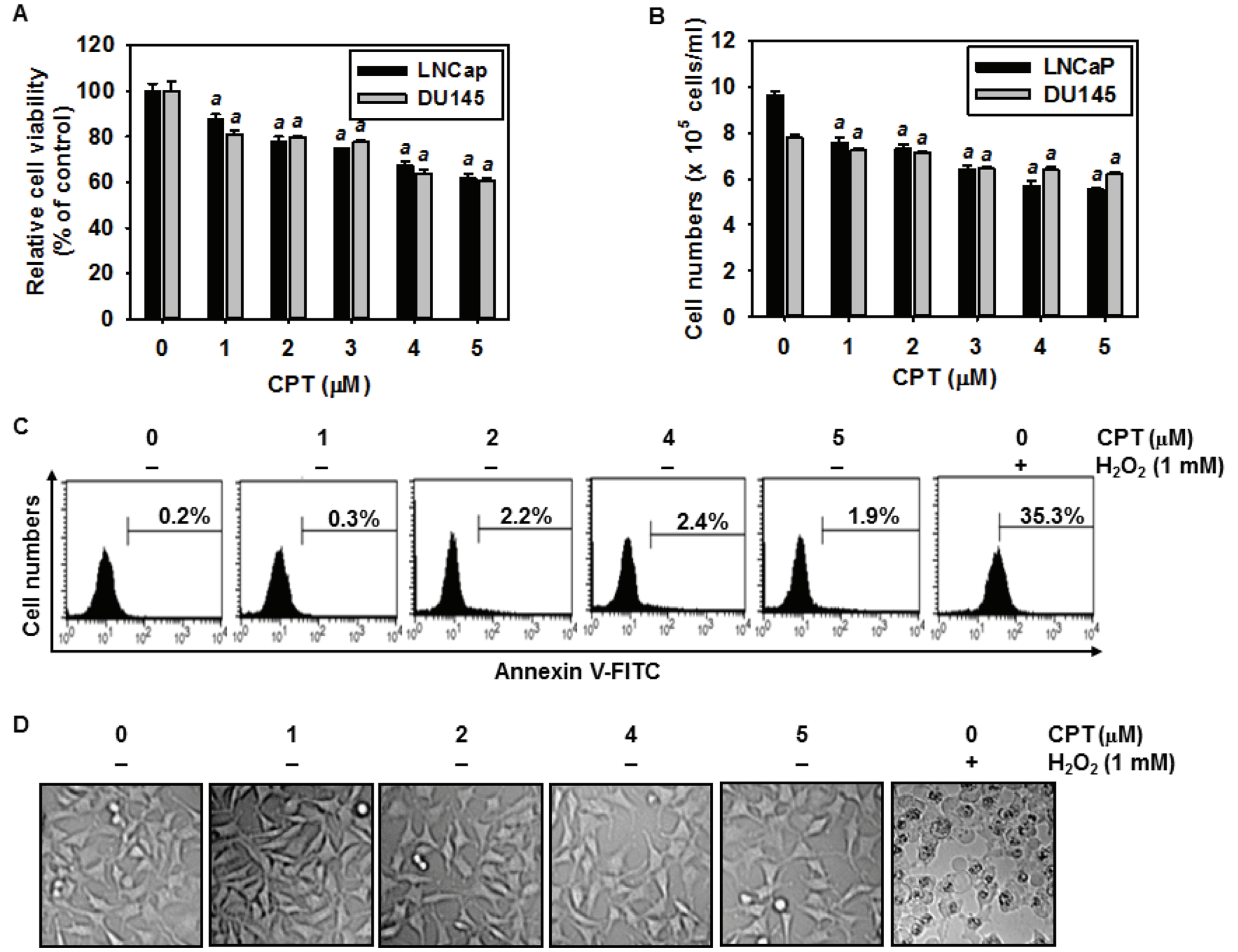

Supplementary Figure 1: Camptothecin (CPT)-decreased cell proliferation in prostate cancer cells. Effect of treatment with CPT on cell viability. Human prostate cancer LNCaP and DU145 cells were treated with CPT at the indicated concentrations for 24 h. (**A**) MTT assay to measure cellular viability. (**B**) In a parallel experiment, cell number was counted using hemocytometer counts of trypan blue-excluding cells. (**C**) The cells were stained with annexin V^+ and analyzed by flow cytometry. (**D**) The cellular morphology was examined by light microscopy. Data from three independent experiments are shown as the overall mean ± S.E. Statistical significance was determined by one-way ANOVA ($^{a}p < 0.05$ *vs*. untreated control).

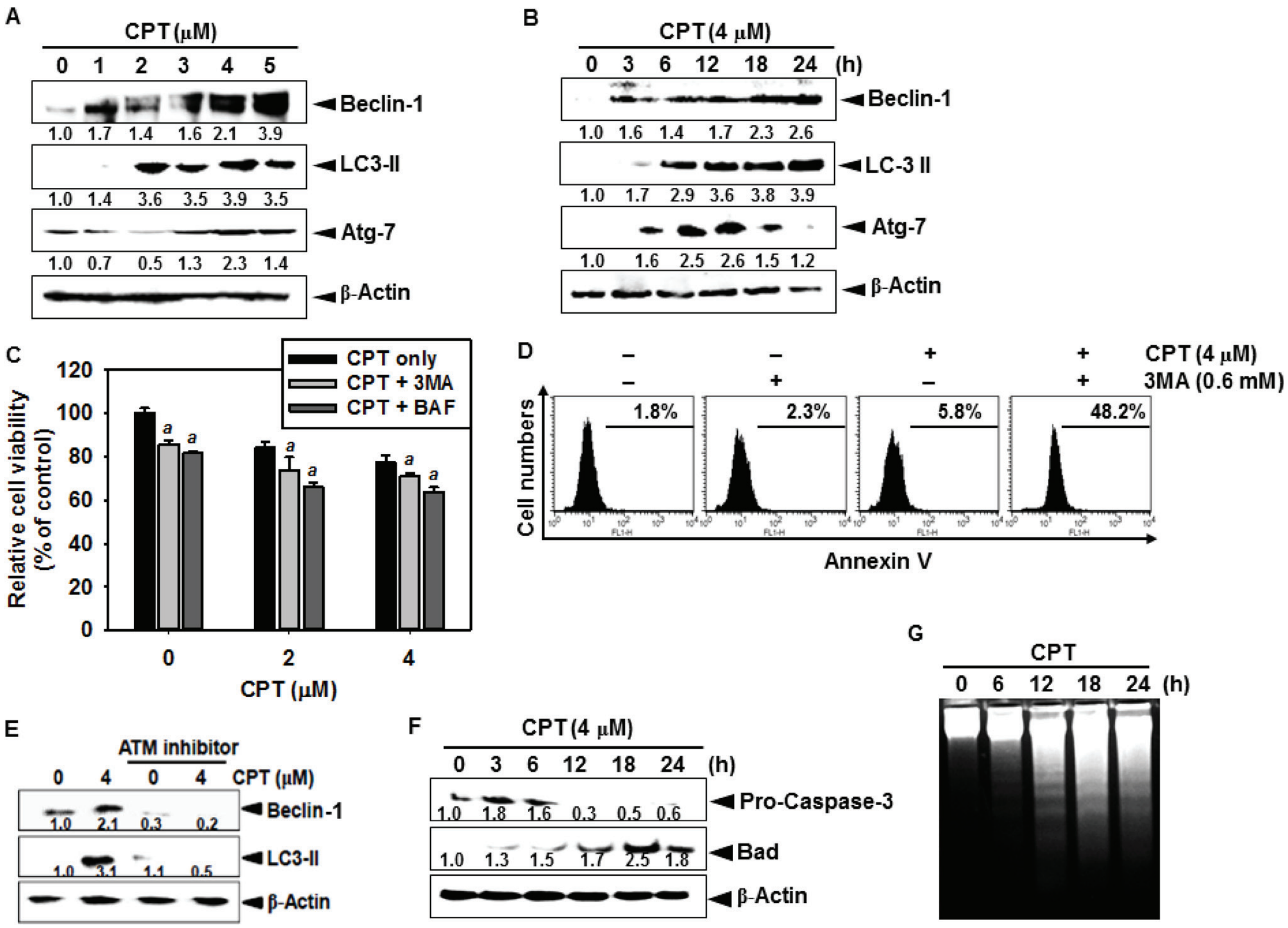

Supplementary Figure 2: Camptothecin (CPT)-induced autophagy as a cytoprotective mechanism in LNCaP cells. LNCaP cells were treated with CPT at the indicated concentrations for 24 h (**A**) and 4 μM CPT at the indicated time points (**B**). Cell lysates were resolved on SDS-polyacrylamide gels, transferred to nitrocellulose membranes and probes with antibodies against Beclin-1, LC3-II, and Atg-7. β-Actin was used as an internal control for western blot analysis. (**C**) LNCaP cells were pretreated with autophagy inhibitors, 0.6 mM 3-methyladenine (3MA) and 20 nM bafilomycin (BAF) prior to incubation with 4 μM CPT for 24 h. Cellular viability was assessed by MTT treatment. (**D**) LNCaP cells were pretreated with 3MA and the cells were stained with annexin V^+ and analyzed by flow cytometry. (**E**) Effect of an ATM inhibitor on CPT-induced autophagy. LNCaP cells were pretreated with the ATM inhibitor prior to incubate with 4 μM CPT for 24 h. Total protein was subjected to 10% SDS-PAGE followed by western blot analysis with antibodies specific for phosphorylated forms of Beclin-1 and LC3-II. (**F**) CPT induces apoptosis in U937 leukemia cancer cells. U937cells were treated with 4 μM CPT at the indicated time points. Cell lysates were resolved on SDS-polyacrylamide gels, transferred to nitrocellulose membranes and probes with antibodies against caspases-3 and Bad. (**G**) In a parallel experiment, fragmented DNAs were isolated from the U937 cells and analyzed on 1.5% agarose gel. Data from three independent experiments are shown as the overall mean ± S.E. Statistical significance was determined by one-way ANOVA ($^a p < 0.05$ *vs*. each CPT-treated control).
